# IGF2BP1 knockdown associates with alternative splicing changes of transcription factor genes in neuroblastoma cells

**DOI:** 10.3389/fonc.2026.1912388

**Published:** 2026-10-20

**Authors:** Jiwen Cheng, Pu Zhao, Junpeng Hui, Ping Cao

**Affiliations:** 1 Department of Pediatrics, First Affiliated Hospital of Xi’an Jiaotong University, Xi’an, Shaanxi, China; 2 Department of Neonatology, Shaanxi Provincial People’s Hospital, Xi’an, Shaanxi, China; 3 Department of Pathology, Xi’an Children’s Hospital, Xi’an, Shaanxi, China; 4 Department of Pediatric Surgery, the Second Affiliated Hospital of Xi’an Jiaotong University, Xi’an, Shaanxi, China

**Keywords:** alternative splicing, IGF2BP1, neuroblastoma, RNA-seq transcriptome, transcription factor

## Abstract

**Background:**

IGF2BP1 is an oncofetal RNA-binding protein with oncogenic functions in neuroblastoma, but whether IGF2BP1 knockdown is associated with alternative splicing (AS) changes of transcription factor (TF) genes remains unclear.

**Methods:**

We re-analyzed RNA-seq data from IGF2BP1-knockdown BE(2)-C cells (three knockdown and three control samples). Differentially expressed genes (DEGs) and AS events (ASEs) were identified, followed by GO/KEGG enrichment. Differentially spliced genes were intersected with a human TF list. Isoform-specific qRT-PCR was performed in control and IGF2BP1-knockdown BE(2)-C cells to validate PSI changes. RIP-qPCR was used to test binding of IGF2BP1 to candidate TF mRNAs. ENCODE eCLIP-seq data from HepG2 and K562 cells were analyzed as indirect supportive evidence.

**Results:**

We identified 807 DEGs and 3, 053 significant ASEs. Among genes with significant splicing changes, 64 were annotated as TFs; 20 splicing events corresponding to 18 genes passed stringent filtering. Independent qRT-PCR validated the PSI changes of LMO4 (A5SS), TCF7L2 (cassette exon), and EHMT2 (exon skipping), which correlated with RNA-seq predictions. RIP-qPCR confirmed that IGF2BP1 binds to LMO4, TCF7L2, and EHMT2 mRNAs in cells, but not to NCOR2. eCLIP-seq data from non-neuroblastoma cell lines provided indirect supporting evidence.

**Conclusion:**

IGF2BP1 knockdown is associated with widespread AS changes of TF genes in neuroblastoma cells, and IGF2BP1 binds to LMO4, TCF7L2, and EHMT2 mRNAs. These findings provide a correlative resource and hypotheses for future mechanistic studies; direct causality remains to be established.

## Introduction

Neuroblastoma is the most common extracranial solid tumor in children and shows marked clinical heterogeneity, ranging from spontaneous regression to aggressive progression (1). Despite intensive multimodal therapy, survival for high-risk patients remains below 40% (2). Compared with adult tumors, neuroblastoma has fewer gene mutations but widespread chromosomal aberrations and epigenetic abnormalities (3). Recent studies highlight the critical role of post-transcriptional dysregulation, especially alternative splicing mediated by RNA-binding proteins, in tumorigenesis (4).

Transcription factors are core regulators of gene expression and are themselves subject to multilayered regulation. About 20% of known oncogenes encode transcription factors, yet direct therapeutic targeting of transcription factors remains limited (5). Alternative splicing greatly expands proteomic diversity by generating multiple functionally distinct isoforms from a single gene (6). In neuroblastoma, aberrant splicing events affect key gene functions; for example, isoform switching of WT1 and TrkA alters their pro- or anti-tumorigenic activities (7, 8).

IGF2BP1 is an oncofetal RNA-binding protein that functions as an m6A reader and regulates stability, localization, and translation of target mRNAs (9). It is highly expressed in various cancers and promotes proliferation, invasion, and chemoresistance (10). In neuroblastoma, IGF2BP1 expression correlates with MYCN amplification and poor prognosis (11). Recent evidence suggests that HNRNPA2B1 and HNRNPR stabilize ASCL1 in an m6A-dependent manner (12). However, it remains unclear whether IGF2BP1 knockdown is associated with alternative splicing changes in neuroblastoma and how such changes might correlate with transcription factor function. This study systematically investigated the association between IGF2BP1 knockdown and alternative splicing patterns of TF genes in neuroblastoma.

## Materials and methods

### Public data acquisition and processing

Raw RNA-seq data were obtained from GEO (GSE161086; Glaß et al., 2021) (13), comprising three IGF2BP1-knockdown BE(2)-C samples and three control samples (transfected with control siRNA). We used SRA Toolkit (v2.10.9) to convert.sra to fastq, filtered low-quality reads with FASTX-Toolkit (v0.0.13) (-q 20 -p 90), and assessed clean reads with FastQC (v0.11.9). No explicit batch information was provided for GSE161086; all samples were processed in the same laboratory and were therefore treated as a single batch. Sample-level PCA based on global gene expression showed separation by experimental group rather than batch (**Supplementary Figure S1**). In contrast, all qRT-PCR, RIP-qPCR, and Western blot experiments in this study were performed independently in our laboratory using an independent IGF2BP1 knockdown.Sequence alignment and differential expression analysis.

We aligned clean reads to GRCh38/hg38 using HISAT2 (v2.2.1) (max 4 mismatches), sorted and indexed alignments with SAMtools (v1.15), and quantified gene reads with featureCounts (v2.0.1) based on GENCODE v38 annotation. FPKM values were calculated for visualization purposes only (e.g., heatmaps and boxplots) and were not used for statistical testing. Differential expression analysis was performed using DESeq2 (v1.34.0) on raw counts. Significant DEGs met |log2FC| ≥ 1 and Benjamini-Hochberg-adjusted FDR < 0.05.

### Alternative splicing analysis

We detected and quantified alternative splicing events using ABLAs (v1.0) (14), which identifies ten splicing types: ES, A5SS, A3SS, IR, MXE, 5pMXE, 3pMXE, CE, and composite events (A3SS&ES, A5SS&ES). For reliable quantification, we retained events with ≥10 splice junction reads in ≥80% of samples. We used Fisher’s exact test to compare groups, considering events significant if the adjusted P < 0.05. In addition, we applied an effect-size threshold (|ΔPSI| ≥ 0.10) and required cross-sample consistency. We did not perform gene-level FDR correction.

### CLIP-seq data analysis

We downloaded eCLIP-seq data for IGF2BP1 in K562 and HepG2 cells from ENCODE (ENCSR062UWG, ENCSR405TAI). The BED files represent significantly enriched binding peaks. We mapped peaks to GRCh38/hg38 and categorized them into 5’UTR, 3’UTR, CDS, nc_exon, intron, antisense, and intergenic regions. Genes with peaks in gene bodies or UTRs were defined as potential IGF2BP1 targets.

### Cell culture

The human neuroblastoma BE(2)-C cell line was purchased from the American Type Culture Collection (ATCC, catalog number CRL-2268, Manassas, VA, USA). Cells were cultured in a 1:1 mixture of Dulbecco’s modified Eagle’s medium (DMEM) and Ham’s F12 nutrient mixture (Gibco, Thermo Fisher Scientific, Waltham, MA, USA) supplemented with 10% fetal bovine serum (FBS, Gibco) and 1% penicillin/streptomycin (Gibco) at 37 °C in a humidified atmosphere containing 5% CO2.

### IGF2BP1 knockdown in BE(2)-C cells

BE(2)-C cells were transfected with IGF2BP1-specific siRNA (si-IGF2BP1) or negative control siRNA (si-Ctrl) using Lipofectamine RNAiMAX (Invitrogen) according to the manufacturer’s instructions. The final siRNA concentration was 25 nM. Both siRNAs were synthesized by Sangon Biotech (Shanghai, China). The IGF2BP1-specific siRNA target sequence was 5′-GGACUUGGAGAAAGUGUUUTT-3′. A non-targeting siRNA (si-NC: 5′-UUCUCCGAACGUGUCACGUTT-3′) was used as a negative control. Cells were harvested 72 h after transfection. This siRNA sequence was independent of the siRNA pool used in Glaß et al. (2021), providing independent replication of the knockdown effect. Knockdown efficiency was verified by Western blotting (**Supplementary Figure S6**).

### qRT-PCR validation of alternative splicing

To independently validate the RNA-seq predictions, total RNA was extracted from control (si-Ctrl) and IGF2BP1-knockdown (si-IGF2BP1) BE(2)-C cells (n = 3 per group) from an independent knockdown experiment using TRIzol reagent. cDNA was synthesized using HiScript III RT SuperMix (Vazyme). For LMO4 A5SS, isoform-specific primers were designed to distinguish the proximal and distal 5’ splice site variants. For TCF7L2 (cassette exon) and EHMT2 (exon skipping), primers were designed to amplify the inclusion and skipping isoforms, with at least one primer spanning the exon-exon junction to avoid genomic DNA amplification. qRT-PCR was performed using SYBR Green chemistry. For LMO4 A5SS, PSI was calculated as: PSI = [proximal isoform]/([proximal isoform] + [distal isoform]) × 100%. For TCF7L2 and EHMT2, PSI was calculated as: PSI = [inclusion isoform]/([inclusion isoform] + [skipping isoform]) × 100%. The relative abundance of each isoform was determined by 2^-ΔΔCt^ normalized to GAPDH. All reactions were performed in triplicate, and three independent biological replicates were analyzed. Primer sequences are listed in **Supplementary Table S1**.

### RNA immunoprecipitation-qPCR

RIP was performed using the Magna RIP RNA-Binding Protein Immunoprecipitation Kit (17-700, Millipore) according to the manufacturer’s guidelines. In brief, BE(2)-C cells were lysed in RIP lysis buffer supplemented with a protease inhibitor cocktail (Roche) and RNase inhibitor (SUPERase-In, Invitrogen). The cell lysate was incubated with 5 μg of anti-IGF2BP1 antibody (ab184305, Abcam) or normal mouse IgG (negative control), both pre-bound to protein A/G magnetic beads (Thermo Fisher), overnight at 4 °C under gentle rotation. Following immunoprecipitation, the beads were washed extensively. An aliquot of the bead-protein complexes was reserved for Western blotting to validate IGF2BP1 pulldown, and the remaining beads were used for RNA isolation. RNA was isolated using TRIzol reagent and reverse-transcribed into cDNA. Enrichment of specific target mRNAs (LMO4, TCF7L2, EHMT2, and NCOR2) was quantified via qPCR using SYBR Green chemistry, with GAPDH as a negative control. Enrichment was calculated as %Input after normalization. The Ct values for IgG controls ranged from 32.5 to 34.8 (mean ± SD: 33.6 ± 0.8). Specific enrichment was defined as fold enrichment over IgG greater than 2. Primers were designed within constitutive exons and do not distinguish between pre-mRNA and mature mRNA; therefore, the RIP assay may enrich both. Primer sequences are listed in **Supplementary Table S1**.

### Western blot validation of RIP

The reserved bead-protein complexes, along with Input (total cell lysate) and IgG IP, were boiled in SDS loading buffer, separated by SDS-PAGE, and transferred to PVDF membranes. After blocking with 5% non-fat milk, membranes were incubated with anti-IGF2BP1 antibody (ab184305, Abcam, 1:1000) overnight at 4 °C, followed by HRP-conjugated secondary antibody. Immunoreactive bands were visualized using enhanced chemiluminescence (ECL).

### Functional enrichment analysis

We performed GO and KEGG enrichment analyses for DEGs and ASE-associated genes using KOBAS 2.0 with hypergeometric testing and Benjamini-Hochberg correction (FDR < 0.05) (15).

### Transcription factor analysis

We downloaded the human transcription factor (TF) list from TRRUST v2 (https://www.grnpedia.org/trrust/, accessed March 2025). We intersected significantly alternatively spliced genes with this TF list to identify TFs with differential splicing. We refer to these as “annotated TFs” rather than validated regulators.

### Statistical analysis

Statistical analyses were performed using R (v4.1.2). Two-group comparisons used two-tailed Student’s t-test; paired t-test where applicable. Multiple-group comparisons used one-way ANOVA followed by Tukey’s HSD test. PCA used factoextra (v1.0.7); heatmaps used pheatmap (v1.0.12). Genomic visualization used Gviz (v1.38.0) and IGV (v2.11.9). For multiple comparisons in alternative splicing analyses, P-values were adjusted using the Benjamini-Hochberg method. P < 0.05 was considered statistically significant.

## Results

### IGF2BP1 knockdown remodeled the BE(2)-C transcriptome, and its expression correlated with neuroblastoma progression

To investigate the transcriptional role of IGF2BP1 in neuroblastoma, we reanalyzed GSE161086 (three IGF2BP1-knockdown BE(2)-C samples and three controls) (13). Sample clustering heatmap showed clear separation between knockdown and control groups (**Figure 1A**), indicating effective transcriptome remodeling. Using |log2FC| ≥ 1 and FDR < 0.05, we identified 807 DEGs: 495 upregulated and 312 downregulated after IGF2BP1 knockdown (**Figures 1B, C**). GO biological process analysis showed that upregulated genes were enriched in neuronal function and cell motility (e.g., cilium movement, cell projection organization, axoneme assembly) (**Figure 1D**), whereas downregulated genes were enriched in developmental and metabolic processes (e.g., angiogenesis, nervous system development, carbohydrate metabolism) (**Figure 1E**).

**Figure 1:**
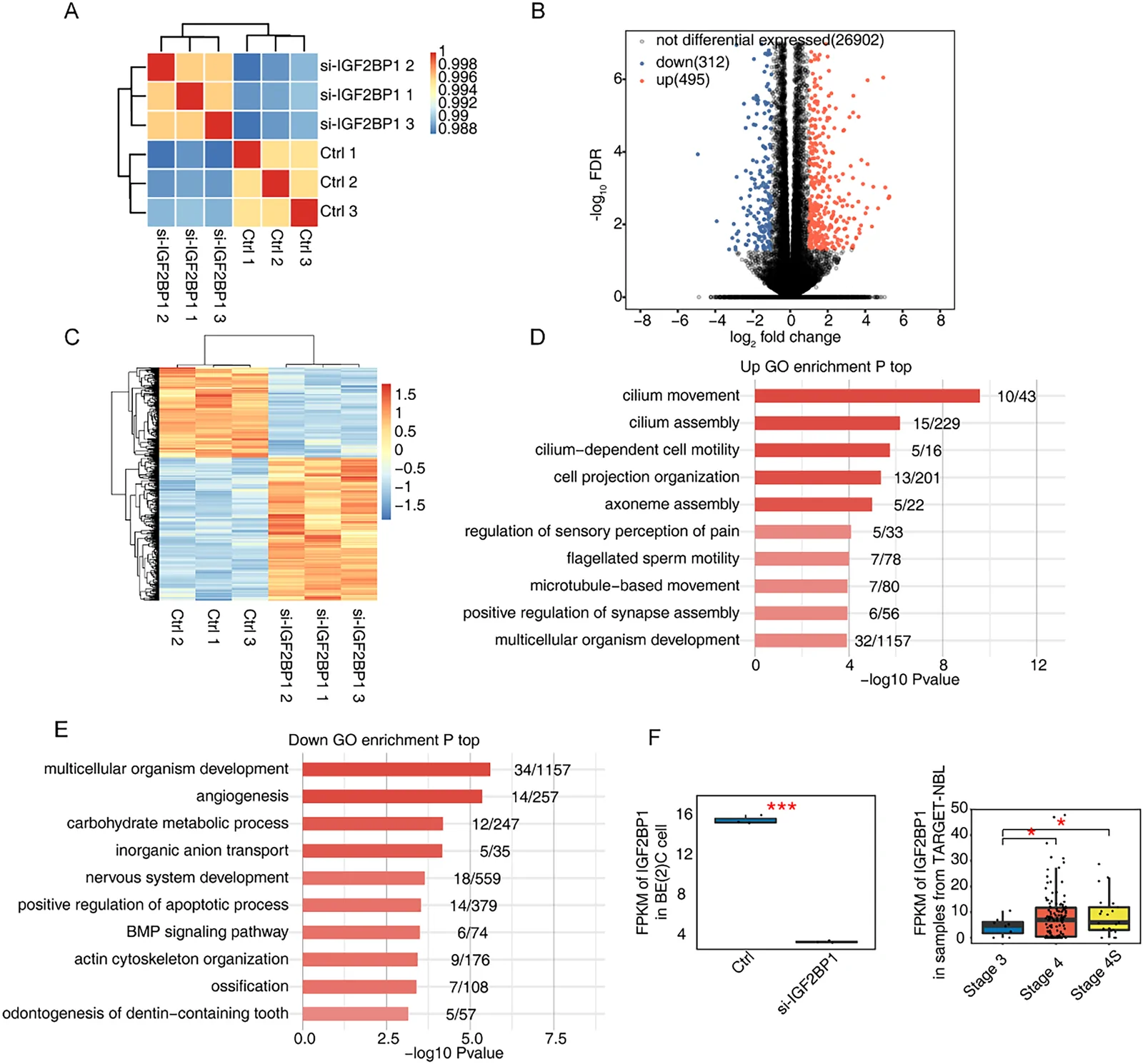
Transcriptome analysis of differentially expressed genes in IGF2BP1-knockdown (si-IGF2BP1) and control (Ctrl) BE(2)-C cells. **(A)** Hierarchical clustering heatmap showing correlations between samples based on FPKM values of all expressed genes. **(B)** Volcano plot of all DEGs (FDR ≤ 0.05, |log_2_FC| ≥ 1). **(C)** Heatmap of expression profiles of all DEGs. **(D)** GO biological process terms enriched among upregulated DEGs. **(E)** GO terms enriched among downregulated DEGs. **(F)** Boxplot showing IGF2BP1 expression in BE(2)-C cells (si-IGF2BP1 vs. Ctrl) and across different stages in the TARGET neuroblastoma cohort. Data are mean ± SEM; ***P < 0.001, *P < 0.05.

To assess clinical relevance, we analyzed the TARGET neuroblastoma cohort. IGF2BP1 expression increased significantly with advancing tumor stage (**Figure 1F**). KEGG pathway analysis revealed that upregulated genes were enriched in neuroactive ligand-receptor interaction (**Supplementary Figure S2A**), while downregulated genes were enriched in glycan biosynthesis and PI3K-Akt signaling (**Supplementary Figure S2B**).

### IGF2BP1 knockdown associated with widespread alternative splicing changes in BE(2)-C cells

To explore potential post-transcriptional impacts, we analyzed alternative splicing events between knockdown and control cells. We identified nine non-IR splicing types (**Figure 2A**). PCA of reliable events (≥10 junction reads in ≥80% samples) showed distinct clustering of knockdown and control samples (**Figure 2B**).

**Figure 2:**
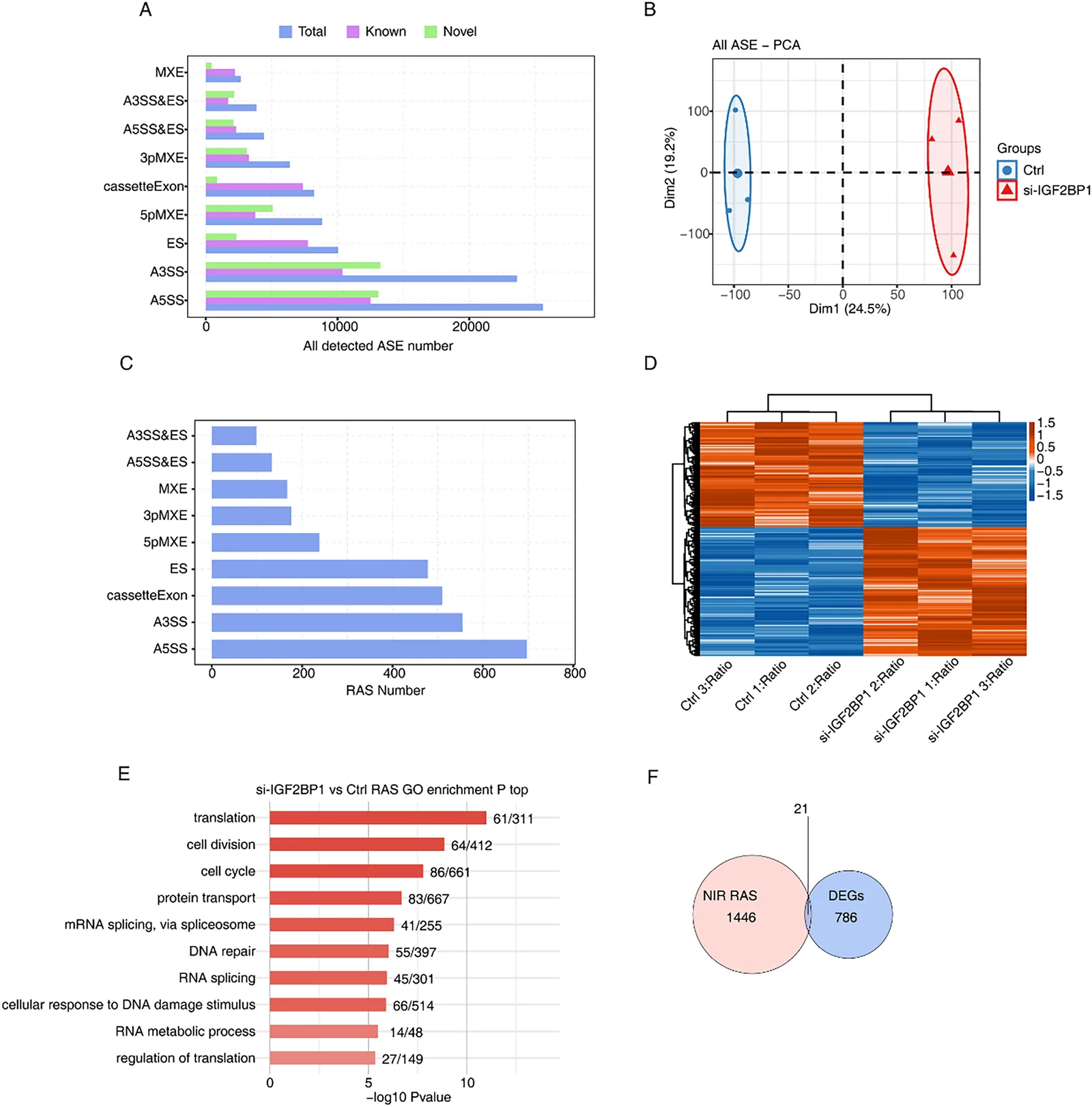
Alternative splicing changes associated with IGF2BP1 knockdown. **(A)** Number of detected ASE types. **(B)** PCA based on PSI values of all ASEs. Confidence ellipses are shown. **(C)** Number of significant differential ASEs (adjusted P < 0.05). **(D)** Hierarchical clustering heatmap of significant differential ASEs. **(E)** GO biological process terms enriched among genes with differential splicing. **(F)** Venn diagram showing overlap between DEGs and differential splicing genes.

Using adjusted P < 0.05, we identified 3, 053 significant differential ASEs across nine types; exon skipping and mutually exclusive exons were most frequent (**Figure 2C**). Hierarchical clustering confirmed that these events clearly separated the two groups (**Figure 2D**).

GO analysis of genes with differential splicing showed enrichment in cell proliferation (cell division, cell cycle) and gene expression regulation (RNA splicing, DNA repair) (**Figure 2E**). KEGG analysis indicated enrichment in metabolic pathways (citrate cycle, purine metabolism) and the spliceosome (**Supplementary Figure S3A**).

Notably, 21 genes overlapped between those with significant splicing changes and DEGs (**Figure 2F**), including known splicing factors and transcriptional regulators (e.g., HNRNPA1, SRSF3). KEGG analysis of these overlapping genes showed enrichment in glucose and lipid metabolism (**Supplementary Figure S3B**).

### IGF2BP1 knockdown associated with alternative splicing changes of transcription factors

To determine whether IGF2BP1 knockdown is associated with TF alternative splicing changes, we examined genes with significant splicing changes. Among the genes with significant splicing changes, 64 were annotated as TFs (**Figure 3A**). Using criteria of ≥10 junction reads in ≥80% samples, we identified 20 splicing events corresponding to 18 genes with reliable splicing changes (**Figure 3B**).

**Figure 3:**
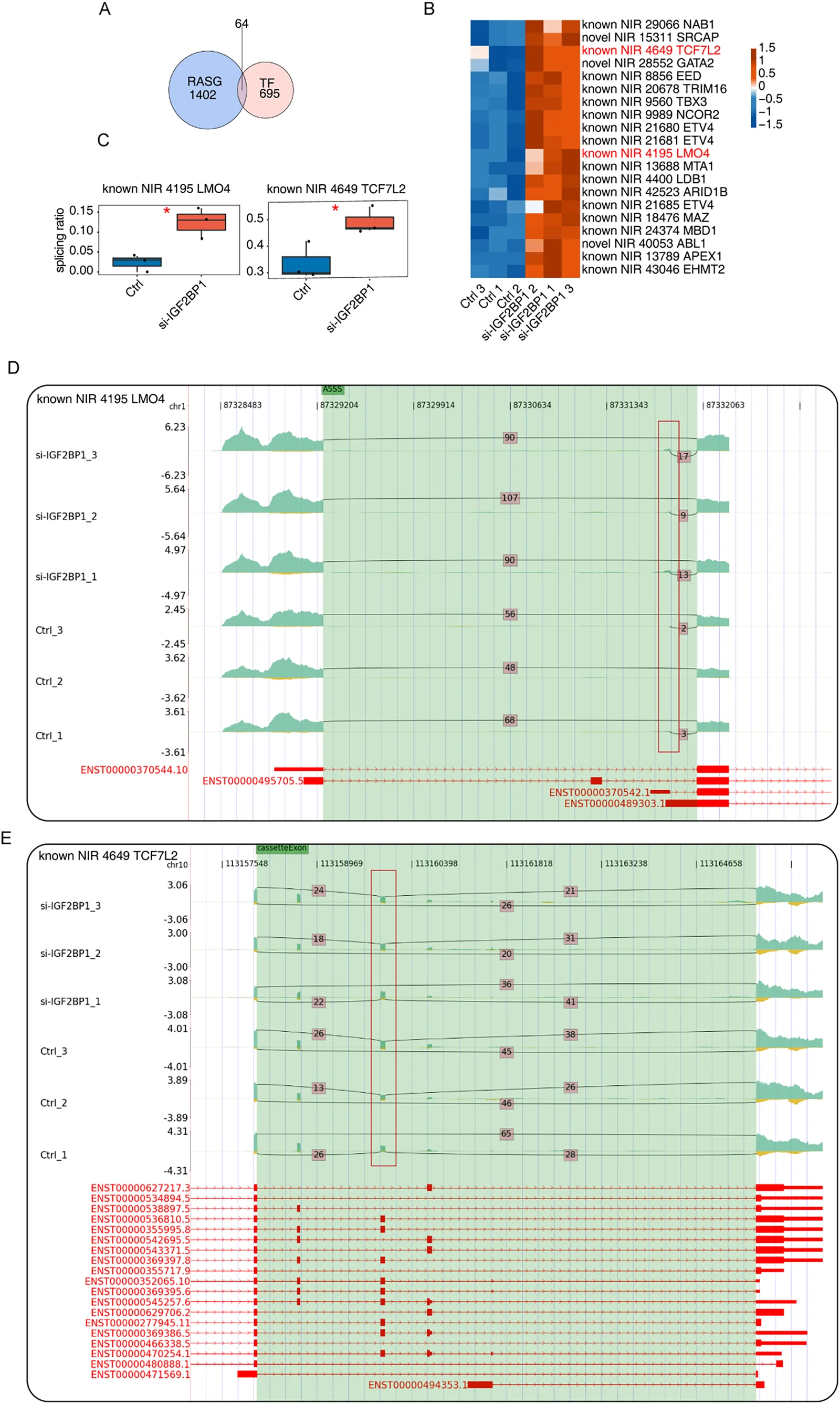
Alternative splicing changes of transcription factors associated with IGF2BP1 knockdown. **(A)** Overlap between differential splicing genes and annotated TFs. **(B)** Heatmap of significant differential splicing events of TFs. Rows represent splicing events; 20 events correspond to 18 genes. **(C)** Boxplot of splicing ratio (PSI) for LMO4 and TCF7L2 events. **(D, E)** IGV visualization of read distributions for LMO4 [**(D)**, A5SS] and TCF7L2 [**(E)**, cassette exon] in control and knockdown samples. Altered exons are marked with red boxes. *P < 0.05.

Alternative splicing of LMO4 and TCF7L2 was altered upon IGF2BP1 knockdown. Based on literature, we focused on LMO4 and TCF7L2, both implicated in neuroblastoma pathogenesis (**Figure 3C**). IGV visualization confirmed distinct splicing patterns between knockdown and control groups for LMO4 and TCF7L2 (**Figures 3D, E**). Additional TFs (EHMT2, NCOR2) were also identified (**Supplementary Figure S4**).

### Independent qRT-PCR validation of alternative splicing changes

To independently validate the RNA-seq predictions, we performed isoform-specific qRT-PCR in si-Ctrl and si-IGF2BP1 BE(2)-C cells from an independent knockdown experiment. IGF2BP1 knockdown significantly increased the PSI of LMO4 A5SS proximal 5′ splice site (42.3% ± 4.1% vs. 67.5% ± 3.8%, ΔPSI = +25.2%, P = 0.003), TCF7L2 alternative exon inclusion (51.2% ± 3.5% vs. 66.8% ± 4.2%, ΔPSI = +15.6%, P = 0.018), and EHMT2 alternative exon inclusion (38.7% ± 4.5% vs. 56.2% ± 3.9%, ΔPSI = +17.5%, P = 0.011) (**Figures 4A–C**). The direction of these PSI changes was consistent with the RNA-seq predictions. Knockdown efficiency was verified by Western blotting (**Supplementary Figure S6**).

**Figure 4:**
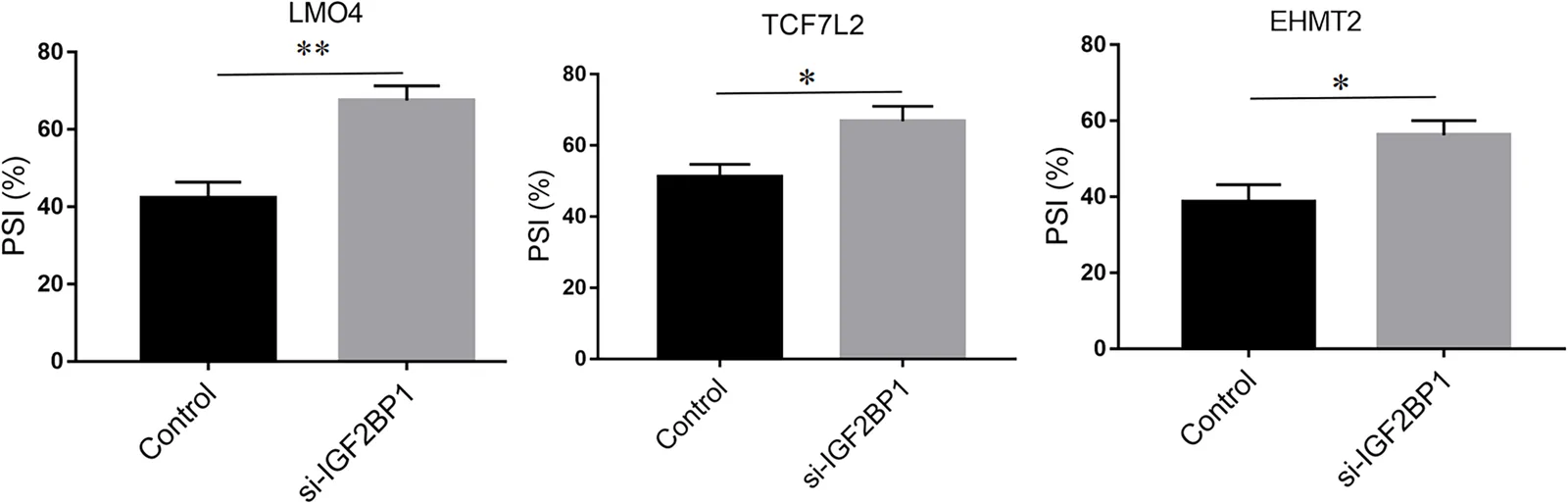
qRT-PCR validation of alternative splicing changes in IGF2BP1-knockdown BE(2)-C cells. **(A–C)** PSI values of the LMO4 A5SS proximal 5′ splice site **(A)**, TCF7L2 alternative exon inclusion **(B)**, and EHMT2 alternative exon inclusion **(C)** in control (Ctrl, n = 3) and IGF2BP1-knockdown (si-IGF2BP1, n = 3) cells. Data are mean ± SEM. **P < 0.01, *P < 0.05 (Student’s t-test).

### IGF2BP1 binds to LMO4, TCF7L2, and EHMT2 mRNAs in BE(2)-C cells

To validate the specificity of IGF2BP1 immunoprecipitation, we first performed Western blotting on Input, anti-IGF2BP1 IP, and IgG IP samples. As shown in **Figure 5A**, IGF2BP1 protein was successfully enriched in the anti-IGF2BP1 IP lane but was absent in the IgG IP lane, confirming the specificity of the antibody. The Input lane showed the presence of IGF2BP1 in total cell lysate.

**Figure 5:**
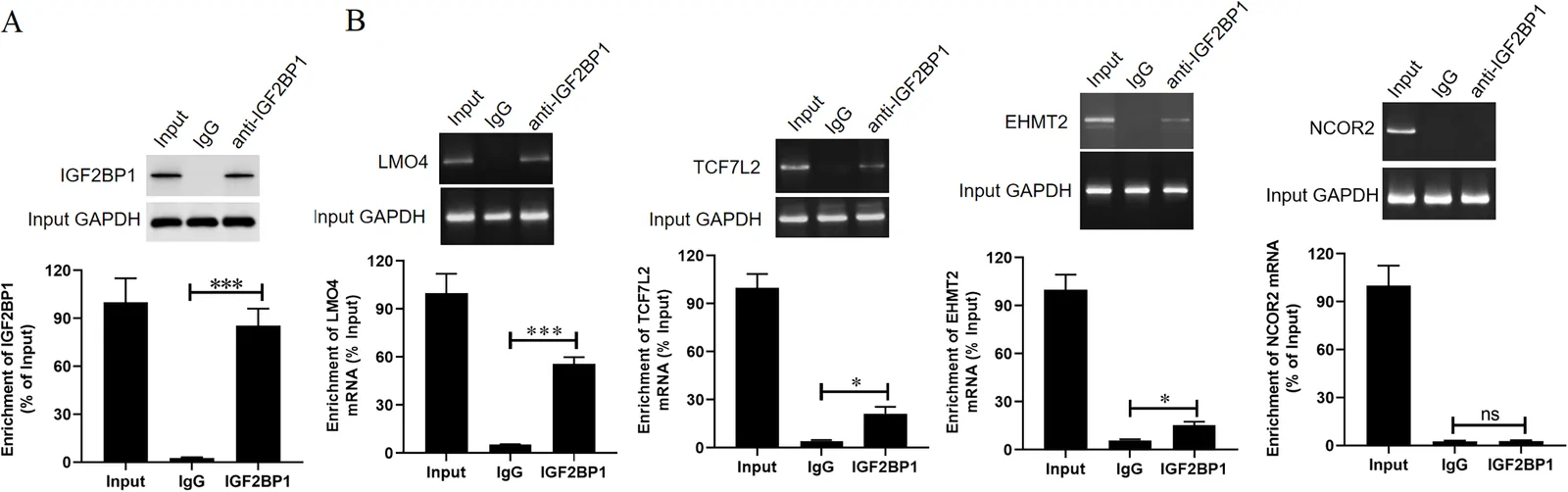
IGF2BP1 binds to LMO4, TCF7L2, and EHMT2 mRNAs in BE(2)-C cells. **(A)** Western blot validation of IGF2BP1 immunoprecipitation. Input, anti-IGF2BP1 IP, and IgG IP were probed with anti-IGF2BP1 antibody. Lower panel: quantification of IGF2BP1 enrichment (% of Input). ***P < 0.001 vs. IgG control. **(B)** RIP-qPCR analysis of LMO4, TCF7L2, EHMT2, and NCOR2 mRNA enrichment. Top: representative gel images of PCR products. Bottom: enrichment expressed as % of Input. GAPDH was used as an internal control for Input normalization. Data are mean ± SEM from three independent experiments. LMO4: ***P < 0.001; TCF7L2 and EHMT2: *P < 0.05; NCOR2: not significant (ns) vs. IgG control. Binding does not imply direct splicing regulation.

We then performed RIP-qPCR to test binding. As shown in **Figure 5B**, Anti-IGF2BP1 antibody significantly enriched LMO4, TCF7L2, and EHMT2 mRNAs (P < 0.001, P < 0.05 and P < 0.05 respectively). No significant enrichment was detected for NCOR2 (P > 0.05), suggesting indirect or cell-type-specific association. These results confirm that IGF2BP1 binds these TF mRNAs. However, whether this binding directly regulates splicing or instead affects mRNA stability/translation remains unknown.

### IGF2BP1 eCLIP-seq binding in non-neuroblastoma cell lines provides indirect supportive evidence

To further examine direct binding, we analyzed ENCODE eCLIP-seq data. In HepG2 cells, IGF2BP1 showed significant binding peaks at LMO4, TCF7L2, and NCOR2 loci (**Figures 6A–D**). In K562 cells, EHMT2 was identified as a bound, alternatively spliced TF (**Supplementary Figure S5**). These data from non-neuroblastoma cell lines provide indirect supporting evidence; direct binding in neuroblastoma was validated by RIP-qPCR (**Figure 5**). The cell-type discrepancy for NCOR2 (bound in HepG2 but not enriched in BE(2)-C RIP-qPCR) suggests cell-type-specific IGF2BP1 binding.

**Figure 6:**
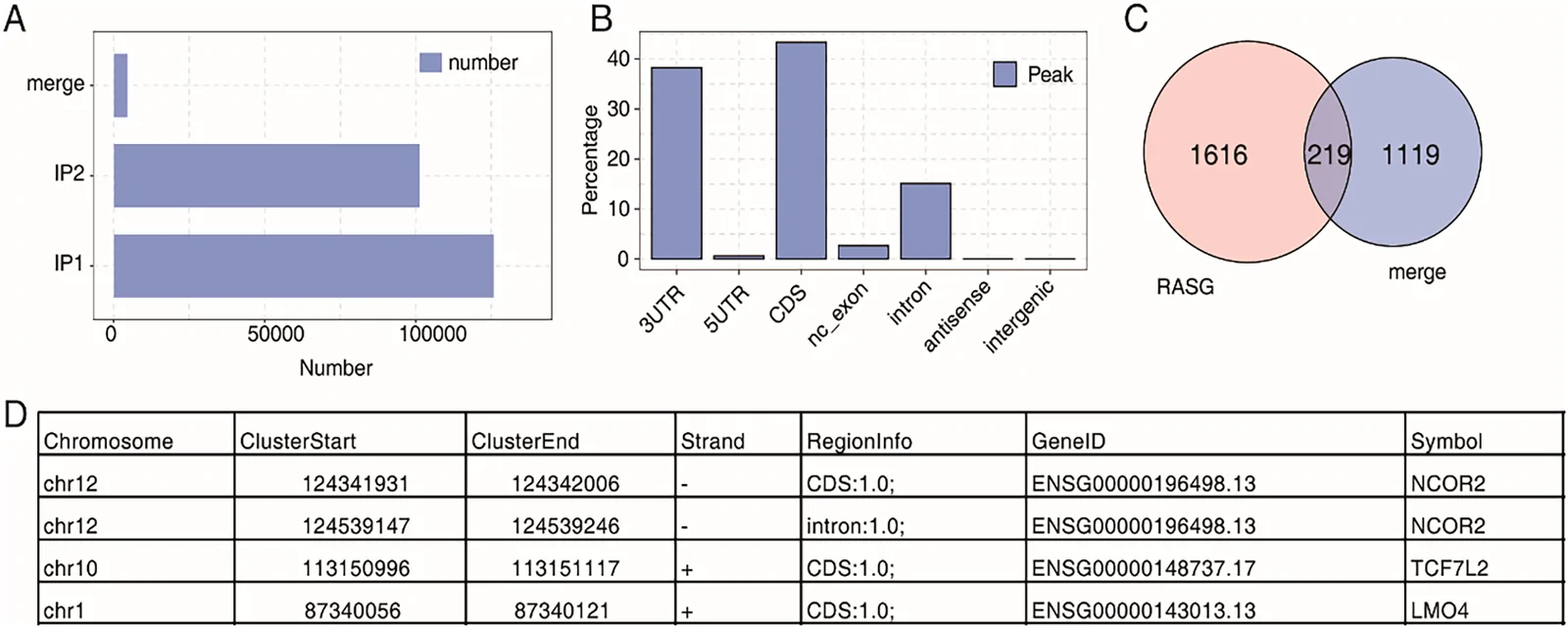
IGF2BP1 eCLIP-seq binding in HepG2 cells (ENCODE ENCSR062UWG). **(A)** Number of IGF2BP1 binding peaks. **(B)** Genomic distribution of peaks. **(C)** Overlap between peak-associated genes and differential splicing genes. **(D)** Peak coordinates of IGF2BP1 binding at NCOR2, TCF7L2, and LMO4 loci.

## Discussion

The RNA-seq dataset GSE161086 was originally generated to investigate IGF2BP1-dependent mRNA stability and E2F-driven gene expression in BE(2)-C neuroblastoma cells (13). That study primarily focused on mRNA turnover and did not systematically analyze alternative splicing. In the present work, we re-analyzed the same dataset with a specific focus on AS changes in transcription factor genes, thereby providing a complementary post-transcriptional perspective. RNA alternative splicing abnormalities are a hallmark of cancer, closely linked to driver gene mutations, tumor heterogeneity, and therapeutic resistance (4). Compared with adult cancers, neuroblastoma has a lower mutation burden but exhibits widespread chromosomal aberrations, epigenetic abnormalities, and post-transcriptional dysregulation. Currently, only a few neuroblastoma-associated splicing events have been reported, such as the isoform switching of WT1 (7) and TrkA (8). In this study, we characterized the alternative splicing landscape associated with IGF2BP1 knockdown in BE(2)-C cells and provided candidate events and hypotheses for future research. It should be emphasized that this study is correlative and does not establish direct causality.

IGF2BP1 is a classic m6A reader, traditionally considered to regulate the stability, localization, and translation of target mRNAs primarily by binding to their 3’UTRs (9). We observed that IGF2BP1 knockdown is accompanied by extensive changes in splicing events, suggesting its potential involvement in broader post-transcriptional regulation. However, RIP-qPCR can only demonstrate binding, not direct splicing regulation. The observed splicing changes may be secondary to altered mRNA stability or translation, or indirectly mediated by changes in the expression of classical splicing factors such as HNRNPA1 and SRSF3. Thus, the present findings should be interpreted as correlative rather than causal.

A central limitation of this study is that we report associations, not direct regulatory mechanisms. Although IGF2BP1 knockdown is associated with splicing changes and IGF2BP1 binds to the corresponding TF mRNAs, we did not demonstrate that this binding directly controls splice site choice. The observed splicing changes could arise from secondary effects, such as altered expression of canonical splicing factors (indeed, we observed changes in HNRNPA1 and SRSF3). Therefore, the observed splicing changes do not establish a direct regulatory mechanism.

Regarding potential mechanisms, only a few unverified hypotheses can currently be proposed. First, IGF2BP1 may cooperate with other RNA-binding proteins or splicing factors to indirectly influence spliceosome assembly or splice site selection. A related mechanism has been reported for other m6A readers: Hu et al. (2024) showed that HNRNPA2B1 and HNRNPR stabilize ASCL1 mRNA in an m6A-dependent manner (12). Whether IGF2BP1 cooperates with these or other splicing-related factors remains to be tested. Second, IGF2BP1 may bind to pre-mRNA and competitively affect the recruitment of splicing factors near variable exons. Third, IGF2BP1 may also indirectly influence splicing through circular RNA or other non-coding RNA networks (16). The public eCLIP-seq data analyzed in this study support that IGF2BP1 can bind to transcripts such as LMO4 and TCF7L2, but these data are from non-neuroblastoma cell lines such as HepG2 and K562 and can only serve as indirect supporting evidence. Whether IGF2BP1 directly regulates splice site choice in neuroblastoma still requires further validation, such as iCLIP to map binding sites at nucleotide resolution, CLIP-qPCR, or minigene assays to test the functional consequence of binding.

The four candidate TFs identified in this study have well-established functions in tumorigenesis. LMO4 is involved in neuronal development, and its high expression is associated with invasive stage IV neuroblastoma; LMO4 activity is essential for neuroblastoma cell invasion (17, 18). TCF7L2 is a Wnt pathway effector, and in neuroblastoma, β-catenin/TCF7L2 can bind to the promoters of numerous Wnt target genes (19). EHMT2 is a histone methyltransferase, and its inhibition can enhance NK cell-mediated anti-tumor immunity (20). As an epigenetic repressor, NCOR2 has been implicated in neuroblastoma differentiation (21), but its broader role in neuroblastoma remains to be fully characterized. The splicing changes of these TFs may alter their transcriptional regulatory programs, but this study did not verify their downstream target genes or functional consequences. Future research could combine transcriptomics, ChIP-seq, or reporter gene experiments to systematically dissect the functional differences of these TF isoforms.

This study has several methodological and interpretive limitations. First, the RNA-seq dataset included only three knockdown and three control samples, and no formal power analysis was performed; although we applied effect-size and cross-sample consistency filtering, we did not perform gene-level FDR correction, so the reported DEGs and ASEs should be considered exploratory. qRT-PCR validated high-effect events but does not exclude false positives among the remaining events. Second, RIP-qPCR confirms binding but not function; we did not perform minigene assays; the RIP-qPCR primers cannot distinguish pre-mRNA from mature mRNA; and we did not test whether splicing changes are secondary to altered splicing factors such as HNRNPA1 and SRSF3. Third, TRRUST annotations include literature-curated and computationally predicted interactions; we did not validate direct transcriptional regulation by these TFs. Fourth, the eCLIP-seq data from non-neuroblastoma cell lines provide supportive but not definitive evidence. Fifth, this study did not validate the clinical significance of these splicing events in an independent clinical cohort.

Future research could delve deeper into the following directions: (1) utilize iCLIP to precisely map IGF2BP1 binding sites in neuroblastoma cells; (2) after mapping the binding sites, conduct LMO4 minigene splicing assays using wild-type and mutant IGF2BP1 binding motifs to test whether IGF2BP1 binding affects A5SS proximal/distal site selection; (3) use CRISPR-mediated endogenous modulation of splicing isoforms (e.g., exon skipping/inclusion for TCF7L2 and EHMT2, and 5′ splice site selection for LMO4) to assess the functional consequences of splicing isoforms, avoiding overexpression artifacts; (4) validate in more neuroblastoma cell lines, organoids, and *in vivo* models; (5) distinguish direct from indirect effects through mediation analysis or splicing factor knockdown/rescue experiments; (6) validate the prognostic or subtype value of candidate splicing events in independent clinical cohorts.

In conclusion, this study shows that IGF2BP1 knockdown is associated with significant alternative splicing changes of transcription factor genes in neuroblastoma cells, and identifies LMO4, TCF7L2, EHMT2, and NCOR2 as candidate splicing-associated transcription factors. IGF2BP1 binds to LMO4, TCF7L2, and EHMT2 mRNAs in cells, supporting an association between IGF2BP1 and post-transcriptional splicing patterns. However, direct causality remains unproven. These findings provide a correlative resource and hypotheses for future mechanistic studies.

## Data Availability

The RNA-seq dataset analyzed in this study is publicly available in GEO under accession number GSE161086 (https://www.ncbi.nlm.nih.gov/geo/query/acc.cgi?acc=GSE161086). ENCODE eCLIP-seq data are available under accession numbers ENCSR062UWG and ENCSR405TAI. TARGET neuroblastoma data are available from the TARGET project at https://ocg.cancer.gov/programs/target. Additional validation data that support the findings are available from the corresponding authors upon reasonable request.

## Supplementary material

Supplementary Figure 1 PCA showed separation by experimental group rather than batch. No batch correction was applied.

Supplementary Figure 2 KEGG pathway enrichment for upregulated **(A)** and downregulated **(B)** DEGs. **(A)** Top 10 enriched KEGG pathways among upregulated DEGs after IGF2BP1 knockdown. **(B)** Top 10 enriched KEGG pathways among downregulated DEGs.

Supplementary Figure 3 KEGG pathway enrichment for differential splicing genes **(A)** and for overlap genes **(B)**. **(A)** KEGG pathways enriched in genes that exhibited differential alternative splicing events upon IGF2BP1 knockdown. **(B)** KEGG pathways enriched in the 21 genes that overlapped between differentially spliced genes and DEGs.

Supplementary Figure 4 Additional transcription factors (EHMT2, NCOR2) showing differential splicing. **(A)** Splicing ratio of EHMT2 and NCOR2 in control (Ctrl) and IGF2BP1-knockdown (si-IGF2BP1) BE(2)-C cells. **(B, C)** IGV visualization of read distributions for EHMT2 **(B)** and NCOR2 **(C)** in control and knockdown samples. Altered exons are marked with red boxes. The y-axis shows normalized read coverage.

Supplementary Figure 5 IGF2BP1 eCLIP-seq binding to EHMT2 in K562 cells (ENCODE ENCSR405TAI). **(A)** Number of IGF2BP1 binding peaks in different eCLIP experiments (merge, IP1, IP2). **(B)** Genomic distribution of IGF2BP1 binding peaks. **(C)** Overlap between IGF2BP1 eCLIP peak-associated genes (merge) and RASG. **(D)** Peak coordinates of IGF2BP1 binding at the EHMT2 locus.

Supplementary Figure 6 Validation of IGF2BP1 knockdown in BE(2)-C cells.BE(2)-C cells were transfected with control siRNA (si-Ctrl) or IGF2BP1-specific siRNA (si-IGF2BP1). Protein lysates were collected 72 h after transfection and analyzed by Western blotting with anti-IGF2BP1 antibody. GAPDH was used as a loading control. Numbers between the IGF2BP1 and GAPDH bands indicate relative IGF2BP1 protein levels normalized to GAPDH.
