# Supplementary material for: IGF2BP1 knockdown associates with alternative splicing changes of transcription factor genes in neuroblastoma cells
Source: Front Oncol. 2026 Oct 20;16:1912388. doi: 10.3389/fonc.2026.1912388 (PMC13727445; doi:10.3389/fonc.2026.1912388)
Supplement: Supplementary file 1 [file Table1.docx]

**Supplementary Table S1. Primers used for isoform‑specific qRT‑PCR validation.**

| Primername | Sequence(5'→3') | Productsize  (bp) | Genomiclocation  (GRCh38) |
| --- | --- | --- | --- |
| LMO4-M-F | GGGAAAAGCAGACCATGGTGA | 91 | chr1:87,329,234-87,329,244; 87,332,012-87,332,021 |
| LMO4-AS-F | CTTAGAAAAACCATGGTGA | 90 | chr1:87,331,805-87,331,814; 87,332,012-87,332,021 |
| LMO4-M/AS-R | CAGCCTGCGCACCGCTTCCA | — | chr1:87,332,072-87,332,091 |
| TCF7L2-M-F | GAGAGACCAATGGAGAAAAA | 86 | chr10:113,158,058-113,158,069; 113,165,554-113,165,561 |
| TCF7L2-AS-F | CGGCCCTTGCAGGAGAAAAA | 86 | chr10:113,159,981-113,159,992; 113,165,554-113,165,561 |
| TCF7L2-M/AS-R | AGCTTCCATCTGAAGAGGGTG | — | chr10:113,165,607-113,165,627 |
| EHMT2-M-F | CCATTCACTCCTCGTGGCT | 98 | chr6:31,889,062-31,890,070; 31,889,227-31,889,235 |
| EHMT2-AS-F | ATTGGACACCCCTCGTGGCT | 99 | chr6:31,888,737-31,888,747; 31,889,227-31,889,235 |
| EHMT2-M/AS-R | AAGAAGAAATGGGCAAAAGACAGC | — | chr6:31,889,291-31,893,314 |

M, reference/main transcript isoform; AS, alternative splicing isoform. M/AS-R indicates a common reverse primer located in a constitutive exon. Genomic coordinates are based on GRCh38/hg38. Product sizes were confirmed by agarose gel electrophoresis. Tm values were calculated using the nearest-neighbor method.
